# Notes from Hospital Practice

**Published:** 1878-04

**Authors:** Henry Banga

**Affiliations:** Chicago


### ALEXIAN BROTHERS' HOSPITAL.

### *A Remarkable Case of Cancer of the Stomach—A Modification of the Stomach Pump.*

Julius Raff, aged 29 years, has always enjoyed good health. Two years ago he left the service in the U. S. army, after a six years' term. Afterward, and up to about two months ago, he held the position of clerk in a country town in Wisconsin. In the beginning of last November he felt sick, complaining of a pressure in the stomach after eating, and vomiting. These symptoms grew worse rapidly, and within two months the strength of the patient was about exhausted, he being reduceed to a mere skeleton. In February, 1878, his friends brought him to Chicago, to the Alexian Brothers' Hospital, under the care of Dr. Mannheimer, with whom I saw the patient the first time, February 11th. From my note book I take the following statement of my first examination: Notwithstanding his great emaciation the patient shows a fair complexion. His mind is clear; he speaks cheerfully about his disease; night-rest undisturbed; there is no fever; pulse 64, and the temperature and moisture of the skin normal. The stomach bears no food; whatever is swallowed is rejected, sometimes soon, but ordinarily from 4 to 6 or 8 hours after eating. The masses which are thrown up consist of half-digested food and decomposed chyle, which are mingled with more or less viscid mucus. The patient has no appetite; he complains of a bad taste and a burning and choking sensation in the throat. The bowels are constipated; fæces dry, hard and gray; the absence of bile in the fæces is the more remarkable, inasmuch as there is no bilious discoloration of the skin or sclerotica. No blood is found in the fæces. The abdomen appears empty and relaxed. In the middle between the xiphoid process of the sternum and the umbilicus, a hard tumor is detected. It has a horizontal direction measuring about two inches, and is about one inch thick. The tumor cannot always be felt, but, at intervals, it comes to the surface, and can then be seen as a distinct prominence at the place described. As soon as the tumor begins moving, the patient suffers great pain, which lasts from one to five minutes. Deep pressure in the epigastric region is always very painful. Lungs and heart and kidneys are in a healthy condition, while the liver is shown, by percussion, smaller than normal. The stomach is undoubtedly greatly dilated, as may be inferred from the extent of the tympanitic resonance, in the left epigastric and hypochondriac regions.

The diagnosis in this case offered some difficulties. Although the presence of the tumor, which evidently belonged to the stomach, suggested that cancer might be the cause of the disorder, yet there were some facts at variance with this supposition, viz.:

1. The age of the patient (29 years).

2. The short space of time—i. e. three months—since the first symptoms suddenly inaugurated the rapid decline of the patient.

3. The absence of any hereditary disposition, as far as this could be ascertained.

4. The relatively fair complexion of the patient. There was no indication of that characteristic cancerous cachexia which, although difficult to describe, is easily recognized by every practitioner.

In the second place we thought of ulcer of the stomach. The age and the absence of symptoms of cachexia would speak in favor of this supposition. On the other hand, the absence of blood in both the fæces and the ejections, would not contradict this diagnosis, since many cases are reported where men have died of gastric disorders, even without showing symptoms of a diseased stomach, and the post mortem revealed perforation by an ulcer as the cause of death.

The symptoms which distressed the patient most—vomiting, eructations, bad taste in the mouth and burning in the throat, occasionally severe pain—were all direct consequences of the gastric catarrh. Therefore sodæ bicarb., magnes. carbon., Karlsbad salt, were prescribed, however with but little or no effect. Antiseptic solutions, as carbolic acid, salicylic acid, salicylate of soda, failed entirely. Morphia alone gave temporary relief from pain. We then resorted to the stomach pump. We introduced the tube for the first time, February 15th. A great quantity of decomposed chyle was removed and the stomach washed with tepid water, afterward with a solution of Karlsbad salt. This operation had a decided effect for the next two days; the patient did not vomit, the burning sensation in the throat entirely ceased, the appetite and digestion were tolerably good, and the paroxysms of pain seemed less frequent. However, on the fourth day vomiting recurred; we again applied the stomach pump and an enormous quantity of decomposed food and mucus was removed. The operation lasted about twenty minutes. Again the effect of washing the stomach was beneficial. The next two or three days the patient felt greatly relieved; he ate and slept well, so that his death, occurring in the afternoon of February 28th, took us rather by surprise.

Unfortunately I was not present at the post mortem, which on the next day was made by Dr. Ernst Schmidt and Dr. Mannheimer. They gave me the following notes: "Beside the extreme emaciation of all organs, there was nowhere any morbid change, except in the stomach. The pyloric portion was transformed into a hard, tense, somewhat nodular tumor; its length measured about 3 inches, while its thickness was from 1½ to 2 inches. It occupied the whole circumference of the pylorus, the opening of which was so narrow as to allow only the passage of a grooved director. The duodenum was relaxed and empty, as was also the remainder of the intestines. The stomach was greatly dilated and distended with gas. It filled the entire left hypochondriac region, the diaphragm being considerably displaced upward. Its contents consisted of gas and a large quantity of decomposed fluid. The liver was smooth, the secretion of bile seemed unimpaired. The tumor was not connected with the adjoining organs; its surface was smooth. Some retroperitoneal glands were enlarged. The tumor, upon dissection, possessed all appearances of a genuine cancer.

The condition of the stomach, as revealed by the autopsy, throws light upon those dark points in the diagnosis, which I have mentioned previously. The extreme stricture of the pyloric portion fully explains what puzzled us so much in the diagnosis of cancer, namely, the short duration of the disorder and the absence of general cachexia. It seems that from the outset the tumor increased in size as an *uniform thickening of the wall of the pylorus*, resulting in constricting its opening. The mere mechanical consequences of the extreme stricture became fatal before cancerous infection of the system took place. *The patient died simply of inanition in consequence of an extreme stricture of the pylorus.*

The apparatus we used for washing out the stomach, was a very simple substitute for the original stomach pump. It consisted of the ordinary stomach tube, to which the one end of a rubber tube, some 5 to 6 feet in length, was attached, while the other end was connected with a funnel or a fountain syringe. Before the tube was passed into the stomach, the vessel was filled with water, and the air expelled from the tube by raising the vessel to fill the tube with water. As soon as the sound was in the stomach, the water was allowed to flow. The higher the vessel was raised, the quicker it emptied itself, and was refilled before the level of the water sank down to the opening, near the bottom of the fountain syringe, in order to avoid aspiration of air. When we thought we had introduced enough water into the stomach, the vessel was put upon the floor, when the water, mixed with the contents of the stomach, would run out. Thus, by alternately refilling and emptying the cavity of the stomach this organ may be thoroughly washed out, till all its contents are removed and the cleansing water returns clear.

The liquid to be used may be tepid water so long as there are contents of the stomach to be removed, after which any medicated mixture the physician deems most appropriate for the mucous membrane, may be substituted. In our case we used for that purpose Karlsbad-water, prepared with the natural Karlsbad-salt, which can be obtained of all druggists. During the operation the patient may remain in bed, in a sitting position, or sit up in a chair. Since the ordinary stomach-pump is a rather expensive and complicated instrument, and therefore not within the reach of every practitioner, it seems to me that the simple and cheap apparatus we used might induce physicians oftener to resort to the procedure of washing out the stomach.

HENRY BANGA, M.D.

CHICAGO, March, 1878.

---

